# Botulinum Toxin Type A Injection for Cervical Dystonia in Adults with Dyskinetic Cerebral Palsy

**DOI:** 10.3390/toxins10050203

**Published:** 2018-05-16

**Authors:** You Gyoung Yi, Keewon Kim, Youbin Yi, Young-Ah Choi, Ja-Ho Leigh, Moon Suk Bang

**Affiliations:** 1 Department of Rehabilitation Medicine, Seoul National University Hospital, Seoul National University College of Medicine, Seoul 03080, Korea; lyk861124@gmail.com (Y.G.Y.); keewonkimm.d@gmail.com (K.K.); sedrinne@gmail.com (Y.Y.); besimple16@naver.com (Y.-A.C.); 2 Department of Rehabilitation Medicine, Incheon St. Mary’s Hospital, Incheon 22711, Korea; mazican@gmail.com

**Keywords:** clinical trials randomized controlled, cervical dystonia, botulinum toxin, dyskinetic cerebral palsy

## Abstract

We aimed to evaluate the efficacy and safety of injecting botulinum toxin A (BoNT-A) into the neck muscles to treat cervical dystonia (CD) in patients with dyskinetic cerebral palsy (CP). This was a randomized, double-blinded, placebo-controlled trial with cross-over design. We prospectively enrolled adults with dyskinetic CP who were over 20 years old and had been clinically diagnosed with CD for more than one year. The primary outcome measure was the change in Toronto Western Spasmodic Torticollis Rating Scale (TWSTRS) at four weeks from the baseline TWSTRS. Seventeen patients were initially enrolled, but one patient was excluded after the final evaluation because of a violation of the study protocol. At four weeks, the BoNT-A injections showed significant improvement in TWSTRS total scores compared to the saline injections (*p* = 0.0286 for ANCOVA). At 12 weeks, the BoNT-A injections resulted in greater improvements in TWSTRS total scores than the saline injections without statistical significance (*p* = 0.0783 for ANCOVA). Dysphagia occurred in three out of 16 patients: two after BoNT-A and one after saline. The dysphagia was transient and improved naturally within two weeks without any special treatment. BoNT-A injection for CD in adults with dyskinetic CP is relatively safe and improves pain and disability.

## 1. Introduction

In adults with dyskinetic cerebral palsy (CP), cervical dystonia (CD) may result in neurologic sequelae such as pain, dysfunction, and cervical myelopathy [1,2,3]. Surgery is often required to ameliorate these symptoms. However, surgical outcomes are not satisfactory because the CD remains untreated even after the surgery. Anticholinergic, antidopaminergic, dopaminergic, and GABAergic medications have been proposed to treat CD in patients with dyskinetic CP, but none have proven to be effective [4]. Previous studies have suggested the botulinum toxin (BoNT) as a primary treatment for idiopathic CD [5,6]. Botulinum toxin A (BoNT-A) has been successfully used for many years in idiopathic CD and is considered the first line of therapy [7]. According to the Cochrane review [7], CD patients with an average baseline Toronto Western Spasmodic Torticollis Rating Scale (TWSTRS) score of 42 points had a decrease of 8.16 points compared with placebo four weeks after BoNT-A injection.

BoNT has also been successfully used in patients with CP for treatment of the upper and lower extremities [8]. However, the effectiveness and safety of BoNT for CD in patients with dyskinetic CP have not yet been fully established. Clinical management of dystonia in these patients is difficult due to abnormal motor engrams that persist from birth, and management is still empirical and largely unsatisfactory [9]. We hypothesized that injection of BoNT-A into the neck muscles of dyskinetic CP patients would reduce the disturbance caused by CD, since pain and impairment are often caused by excessive muscle tone.

The main purpose of this study was to evaluate the efficacy and safety of injecting BoNT-A into the neck muscles of CD in patients with dyskinetic CP. In addition, we investigated whether clinical factors, such as cervical spondylosis, affect the therapeutic effect of BoNT-A injections in these patients.

## 2. Results

Seventeen patients were initially enrolled, but one patient was excluded after the final evaluation because of a violation of the study protocol, which was four times of shoulder and neck injection within the follow-up period after the injection. Demographic and clinical data for the sixteen participants included in the study are presented in Table 1 and the treatment dose and site of injection were presented in Table 2. ANOVA for period effect and the carry-over effect showed that neither effect was significant at a *p*-value of 0.05 (*p* = 0.6740 for period effect and *p* = 0.2097 for carry-over effect).

### 2.1. Changes on the TWSTRS after the Injection

**Figure 1:**
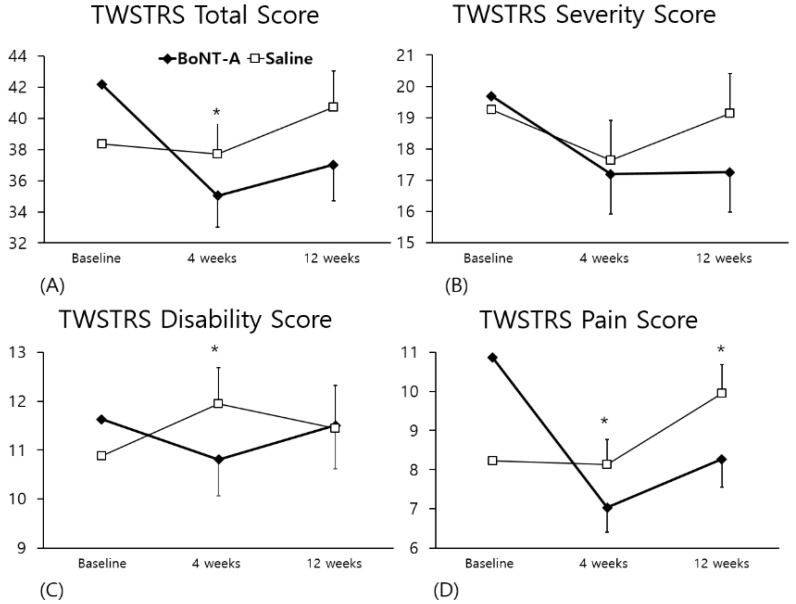
Comparison of the TWSTRS change between the BoNT-A and the Saline injection. * *p* < 0.05 for ANCOVA. The error bar indicates the SE for LS mean change from baseline. BoNT-A Botulinum toxin A, ANCOVA, Analysis of covariance, SE standard error, LS least-squares. (**A**), TWSTRS total score change, scale ranges from 0 to 85, (**B**), TWSTRS severity subscale change, scale range from 0 to 35, (**C**), TWSTRS disability subscale change, scale range from 0 to 30, (**D**), TWSTRS pain subscale change, scale range from 0 to 20, TWSTRS; Toronto Western Spasmodic Torticollis Rating Scale.

TWSTRS total scores at baseline, four weeks, and 12 weeks are presented in Figure 1. At four weeks, the BoNT-A injections showed significant improvement in the TWSTRS total score (Figure 1A) compared to the saline injections (*p* = 0.0286 for ANCOVA). At 12 weeks, the BoNT-A injections had a tendency to show greater improvement than the saline injections, but this difference was not statistically significant (*p* = 0.0783).

There was no significant difference between the two injections on the TWSTRS severity score (Figure 1B). At four weeks there was a significant improvement in the TWSTRS disability score (Figure 1C) for the BoNT-A injections compared to the saline injections (*p* = 0.0152), but there was no significant difference after 12 weeks (*p* = 0.6444). On the TWSTRS pain score (Figure 1D), the BoNT-A injections showed statistically significant improvement compared to the saline injections at both four and 12 weeks (*p* = 0.0013 and 0.0200, respectively).

### 2.2. Changes in Numerical Rating Scale (NRS) after the Injection

**Figure 2:**
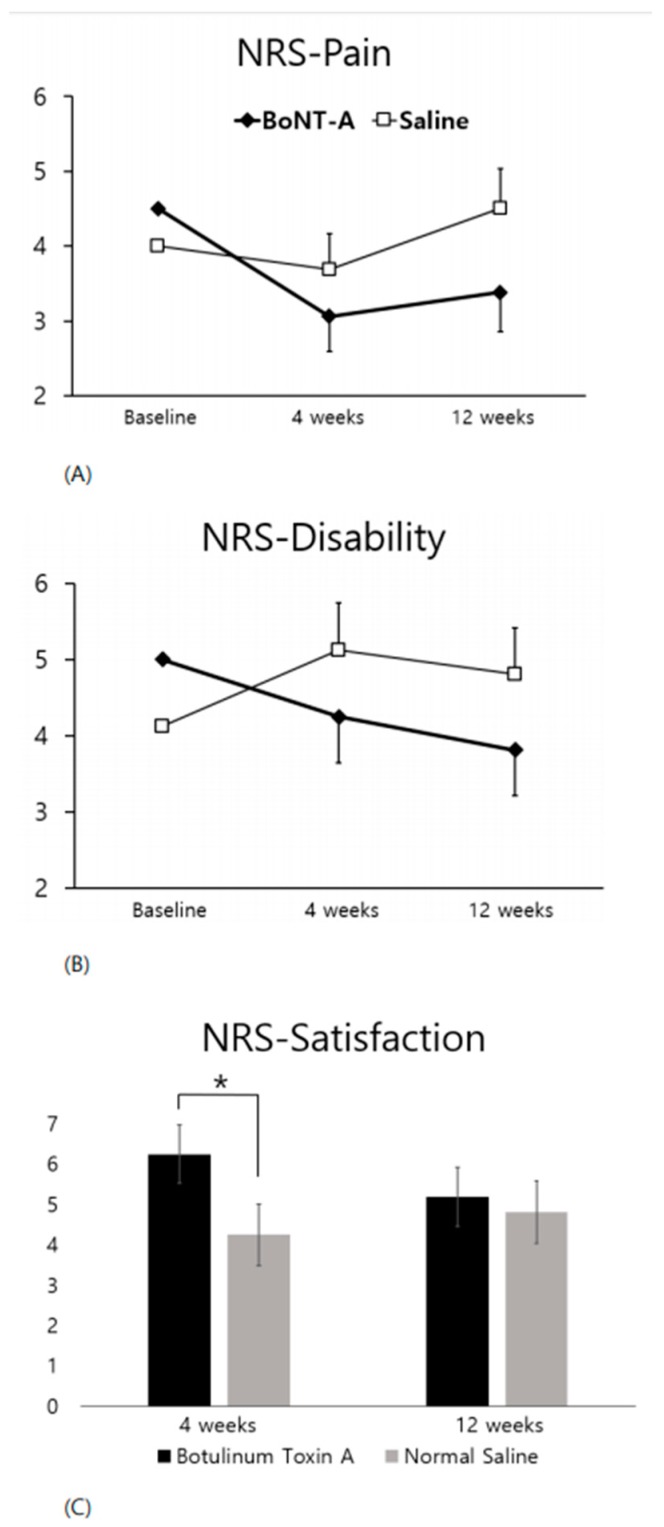
Comparison of NRS scores between the BoNT-A and the saline injection. Comparison of the NRS score at four weeks and 12 weeks between the BoNT-A and the saline injection, * *p* < 0.05 for ANCOVA (**A**,**B**) and Wilcoxon signed-rank test (**C**), In Figure 2A,B, the error bar indicates the SE for LS mean change from baseline. In Figure 2C, the error bar indicates the standard deviation. NRS Numeric rating scale, BoNT-A Botulinum toxin A, ANCOVA, Analysis of covariance, SE standard error, LS least-squares.

Figure 2 presents the NRS for both pain and disability at baseline, four weeks, and 12 weeks and the NRS for satisfaction at four weeks and 12 weeks. On the pain NRS (Figure 2A), the BoNT-A injections had a tendency toward lower pain at four and 12 weeks, but there was no statistically significant difference between the two treatments (*p* = 0.0603 and 0.1796, respectively). On the disability NRS (Figure 2B), the BoNT-A injections had a tendency toward lower disability at four weeks, but there was no statistically significant difference between the two treatments (*p* = 0.1466). On the satisfaction NRS (Figure 2C), which was not obtained at the baseline, the BoNT-A injections scored higher at four weeks and 12 weeks. This difference reached statistical significance only at four weeks (*p* = 0.0176). 

### 2.3. Changes in JOA Score after the Injection

The Japanese Orthopedic Association (JOA) score was constant in most patients. In the BoNT-A group, the average JOA score increased by 0.06 points after four weeks of injection, and the score in the saline group decreased by 0.06 points after four weeks of injection. There was no statistically significant change detected by the ANCOVA between the two groups.

### 2.4. Clinical Factors Associated with the Effect of BoNT-A

**Figure 3:**
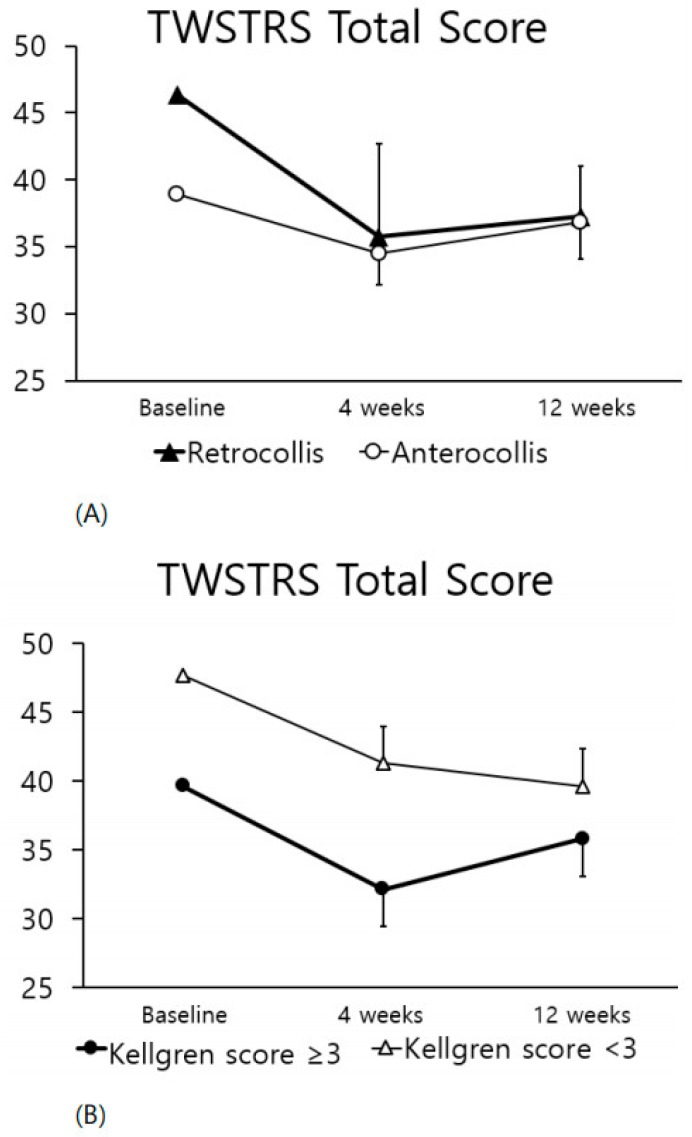
Comparison of the TWSTRS total score change according to clinical factors. Comparison of the TWSTRS total score change at four and 12 weeks according to (**A**) retrocollis or anterocollis; (**B**) maximal cervical spondylosis grade classified using Kellgren score.

When the clinical features of CD included retrocollis rather than anterocollis, the TWSTRS scores showed greater improvement four weeks after injection (Figure 3A), although this difference was not statistically significant. There was no correlation between severity of spondylosis and TWSTRS improvement (Figure 3B) and the two groups (maximal Kellgren grade ≥ 3 or not) showed similar improvement.

### 2.5. Adverse Events

Dysphagia occurred in three of 16 patients, two after the BoNT-A injection and one after the saline injection that may have been caused by altered neck muscle activity and balance of the head and neck posture [10]. Two patients complained of difficulty swallowing and coughing during meals after the BoNT-A injection. In one patient without definite laryngeal aspiration at the baseline videofluoroscopic swallowing study (VFSS), a VFSS performed 10 days after the BoNT-A injection showed laryngeal aspiration of thin liquid (5 mL). In the other patient, laryngeal penetration of post-injection VFSS was observed for thin liquid (5 mL) and curdled yogurt that was not observed in the baseline VFSS. A patient who complained of swallowing difficulty one day after the saline injection showed laryngeal penetration of thin liquid (5 mL) in the post-injection VFSS.

## 3. Discussion

Our study revealed that BoNT-A injections used to treat CD in patients with dyskinetic CP result in greater improvement of TWSTRS scores at four weeks compared with saline injections, especially on the pain and disability subscales.

Previous work concerning BoNT-A injections in CD reported that TWSTRS scores improved in 29 of 37 (78%) patients and were more effective than placebo [11]. One study reported BoNT injections to treat motor symptoms in cases of CD to be effective in 20% to 70% of patients. This study also noted significant improvement in pain and quality of life [12]. BoNT is considered the primary treatment option for CD by reducing sustained involuntary muscle contractions and twitching of the cervical musculature [13,14,15,16]. According to the Cochrane review by Castelao et al. [7], BoNT-A improved the TWSTRS total score and has been reported to be associated with a moderate-to-large improvement in the participant’s baseline clinical status after four weeks. According to a study comparing 55 patients treated with Dysport and 61 patients as a control group, Dysport was reported to be beneficial to CD patients, with an average reduction of TWSTRS scores of 16.8 points in the Dysport group [16]. The mechanism of action of BoNT is to inhibit the release of acetylcholine (ACh) at the neuromuscular junction and block the neuromuscular action potential, which blocks muscle contraction [17,18]. However, in adults with dyskinetic CP, the efficacy and safety of BoNT for the treatment of CD have not been established. 

In patients with dyskinetic CP, failure to receive appropriate early treatment for the dyskinetic hyperactivity may result in the herniation of an intervertebral disc, spondylosis, malalignment, instability of the cervical spine, or a combination of these [2,19]. Pain and disability may also cause structural changes in the neck [2]. Therefore, improvement in pain and function may be effective in reducing the incidence of myelopathy. Patients with dyskinetic CP often require surgery because of myelopathy but often the results of the surgery are not as good as expected due to continued neck movement afterwards [2,3,4,19,20]. In adults with dyskinetic CP, instability and subsequent symptom recurrence are commonly reported [3]. Therefore, it would be helpful to reduce dyskinetic movements with perioperative BoNT injections in order to improve surgical outcomes and prevent the recurrence of myelopathy. 

In the patients with dyskinetic CP who participated in the study, the TWSTRS total score decreased by 7.14 points (17%) at four weeks and 5.16 points (12%) at 12 weeks after BoNT-A injection compared with the baseline score. The TWSTRS severity score decreased by 2.5 points (13%), disability by 0.81 (6%), and pain by 3.83 (35%) at four weeks after the injection. The pain subscale showed the most significant decline with a statistically significant difference. Therefore, BoNT-A injection is helpful in reducing the TWSTRS pain subscale score in dyskinetic CP, contributing to the reduction in the TWSTRS total score (17%), which is slightly less than the 20% reported by Zoons et al. [21] to be clinically relevant in a population with idiopathic CD. In addition, the NRS satisfaction score was 6.27 points at four weeks after injection and was significantly higher than the 4.22 points in the saline group. Taken together, BoNT-A might be a valuable option for CD in patients with dyskinetic CP.

Another study [14] of idiopathic CD reported that the severity score decreased by 6.2 points (30%) during Dysport injection treatment. A similar study reported a 3.9-point decrease (22%) in the severity subscale [16], which was a greater improvement than that observed in the present study (13%). The clinical phenotype differs between patients with idiopathic CD and those with dyskinetic CP having CD. In idiopathic CD, the majority of patients have a combined form of torticollis, laterocollis, with a small proportion of patients having anterocollis and retrocollis. However, the most difficult forms of CD to treat are the combinations with anterocollis and retrocollis, which is the dominant component in patients with dyskinetic CP. The TWSTRS severity score is likely to not be sensitive enough to reflect these changes in patients with dyskinetic CP, as factors related to rotation and laterocollis, as well as anterocollis and retrocollis, account for a large number of points.

There have been many studies of idiopathic CD [13,14,15], but not of CD associated with dyskinetic CP [2,22,23]. In a study of patients with dyskinetic CP [8], botulinum toxin injection for CD was not reported to our current knowledge, but only injected in the muscles of the upper and lower extremities. Recently, the incidence of aging adults with CP has increased due to increased life expectancy. In adults with dyskinetic CP, CD can cause progressive myelopathy as they age. This study is the first to investigate the effect of BoNT injection for CD in adults with dyskinetic CP. The clinical manifestations in this population differ from that of CD in other neurological populations. EMG guidance allowed us to achieve greater clinical improvement by accurately identifying the site of muscle hyperactivity [24]. However, transient dysphagia occurred in two patients after BoNT-A injection. Ultrasound guidance was not used for trapezius and paraspinalis muscles, although sternocleidomastoid, and longissimus were injected with ultrasound guidance in this study. In the case of scalenus (medius and anterior), sono-guided injection was performed only in some cases. According to Hong et al. [25], ultrasound may have prevented dysphagia rather than EMG-guided injection.

When clinical manifestations of CD included retrocollis rather than anterocollis, the TWSTRS score showed a greater improvement at four weeks after injection (Figure 3A). Anterocollis and retrocollis refer to the rotation of the head in the sagittal plane. Anterocollis occurs when the chin is depressed toward the chest, and retrocollis occurs when the chin is lifted and the occiput approaches the back [6,26]. Anterocollis has been reported to be more challenging to treat with BoNT injection than other forms of CD [27,28,29,30,31]. The muscles contributing to anterocollis are the sternocleidomastoid, the scalene muscles, and the longus colli, which are difficult to access for injections [31]. Therefore, the difference in the TWSTRS total score change is thought to be a result of the differences in the ability to inject the muscles in the two presentations although we performed sono-guided injection. In patients with a high Kellgren grade of cervical spondylosis, there was also improvement after the BoNT-A injection (Figure 3B), suggesting that BoNT-A injections can be beneficial for some patients with cervical spine degenerative changes secondary to dyskinetic CP.

There are some limitations to our study. We used a cross-over design due to the small number of dyskinetic CP patients in our research hospital. We failed to recruit 28 patients as initially intended due to the low incidence of this condition. Since there is not a large population of patients with dyskinetic CP, the number of participants we were able to recruit was insufficient for a randomized controlled trial. As patients with dyskinetic CP do not visit tertiary hospitals frequently in South Korea, it was very difficult to recruit patients. Also, there was an absence of long-term follow-up data and we did not observe the effect of regularly applied BoNT injections on pain, disability, spondylosis, and myelopathy in the CD of patients with dyskinetic CP. Lastly, TWSTRS pain and disability scores improved at four weeks after the BoNT-A injection, but not the NRS disability and pain scores. As NRS disability and pain scores are assessed by the patient and the TWSTRS score is determined by the clinician, the TWSTRS score should be more sensitive.

## 4. Conclusions

Botulinum toxin injection for CD in adults with dyskinetic CP is relatively safe and can improve the pain and disability related to CD. 

## 5. Materials and Methods

### 5.1. Study Design

**Figure 4:**
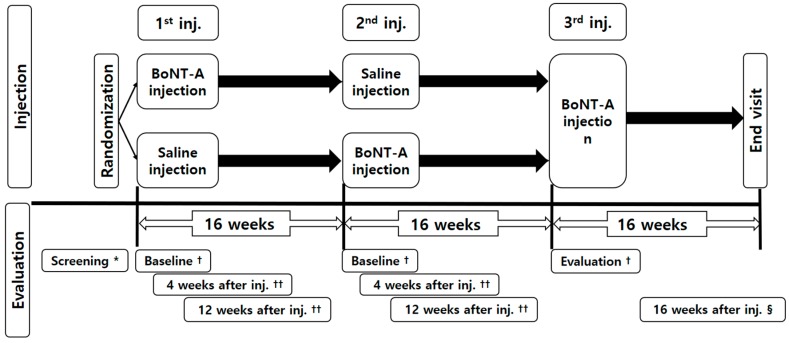
Study flowchart. The first and second injections were included in the analysis; data for the third injection were excluded. * Obtain written consent, evaluation of inclusion/exclusion criteria, demographic surveys, general physical examinations, vital signs, prior medication/history/surgery, pregnancy test, hematologic test, blood chemistry test and urine test, C-spine 3D CT, and adverse reaction investigation. † Vital signs, NRS (except NRS satisfaction score), JOA score, TWSTRS, VFSS, Identification of concomitant medications, and adverse reaction investigation. †† Vital signs, NRS, JOA score, TWSTRS, Identification of concomitant medications, and adverse reaction investigation. § Vital signs, general physical examinations, pregnancy test, hematologic test, blood chemistry test and urine test, NRS, JOA score, TWSTRS, Identification of concomitant medications, and adverse reaction investigation.

This was a prospective, randomized, double-blind, placebo-controlled, cross-over study. Participants were randomly assigned to two groups, and depending on group assignment they were injected either first with BoNT-A and then saline or first saline and then BoNT-A (Figure 4). A third injection of BoNT-A was administered to all patients but the data was not included in the analysis. The participants and the physicians were blind to both the group assignment and to the medication that was being administered so that the participants were free of bias when reporting outcome measures or adverse events. A pharmacist who only knew the randomization code prepared the syringe in liquid form at a pharmacy to keep the physicians blinded. Both the BoNT-A and the saline were provided as liquids in a syringe, and the physician and the patient did not know which was given. The saline may not a true placebo in this study as it does not contain the non-botulinum toxin excipients in the BoNT injections, which could possibly have some activity. A block randomization method was used so that the two groups could be assigned at a 1:1 ratio. 

### 5.2. Standard Protocol Approvals, Registrations, and Patient Consents

This study was registered as a clinical trial (registration number: NCT01860196). All procedures performed in the study were in accordance with the ethical standards of the institutional and/or national research committee and with the 1964 Helsinki declaration and its later amendments or comparable ethical standards. All participants provided written informed consent and the Seoul National University Hospital Institutional Review Board (IRB) approved the study design before participation (IRB No. 1207-028-416).

### 5.3. Participants

We included patients with dyskinetic CP who were over 20 years old and had been clinically diagnosed with CD for more than one year. We excluded the following participants: (1) those who had an allergic reaction to BoNT-A; (2) those with a fixed cervical range of motion limitation; (3) those with uncontrolled symptoms due to fever, infection, malignancy, or uncontrolled epilepsy; (4) women who were pregnant or lactating; (5) those who, at screening, were participating in other clinical trials or were taking muscle relaxants, benzodiazepine, or anticholinergic drugs within four weeks of screening; (6) those who had received a botulinum toxin injection within the previous six months; and (7) those who had been treated with intrathecal baclofen, selective peripheral denervation, or deep brain stimulation. 

### 5.4. Procedure (Interventions)

One vial of BoNT-A (100 U) was diluted in 2 mL of normal saline to a concentration of 5 U per 0.1 mL. The maximum dose given per visit was 200 units. The BoNT-A used in this study was Neuronox^®^, also known in Korea as Meditoxin^®^ (Medytox Inc., Ochang-eup, Cheong-won-gu, Cheongju-si, Chungcheongbuk-do, Republic of Korea). Neuronox^®^ and Meditoxin^®^ are equivalent to BOTOX [32] and have been found to be safe with similar efficacy as has been proven in previous studies in children with CP [33] and in stroke patients [34]. Electromyography was used to record the hyperactivity of the muscle to be injected before starting the injection. The physician set the dose for each muscle to be injected before starting the injection as suggested by Jost and Tatu [35,36], who described BoNT injections for all CD subtypes. Most of the muscles, except the trapezius and the paraspinalis, were injected under ultrasonography-guidance for accurate injection. The injections were performed using an injectable monopolar needle electrode (37 mm, Chalgren, Gilroy, Canada) guided by electromyography (EMG) or ultrasonography. The injections were administered by one of two physicians, each of whom has at least 5 years of experience in using BoNT-A injections for CD. The exact dose and site of the injection were presented in Table 2. 

### 5.5. Assessment

A flowchart of the overall study design is presented in Figure 4. The primary outcome measure was the TWSTRS score at four and 12 weeks compared with baseline. The secondary outcome was the patient’s self-assessment of pain, disability, and satisfaction using a numerical rating scale (NRS) at 4 and 12 weeks compared with baseline. 

### 5.6. Toronto Western Spasmodic Torticollis Rating Scale Score

The Toronto Western Spasmodic Torticollis Rating Scale (TWSTRS, range 0–85) consists of three subscales that grade the severity (range 0–35), disability (range 0–30), and pain (range 0–20) of the CD.

### 5.7. Numerical Rating Scale

Patients rated their pain, disability, and satisfaction using the Numerical Rating Scale (NRS). The NRS pain score was a scale designed such that the far left (0) indicated “no pain” and the far right (10) indicated the “worst pain imaginable”. Similarly, on the NRS disability score, the far left (0) indicated “no disability” and the far right (10) indicated the “worst disability”. On the NRS satisfaction score, which was not applicable to the baseline evaluation, the far left (0) indicated “no satisfaction” and the far right (10) indicated the patient was “very satisfied.”

### 5.8. Japanese Orthopedic Association Score 

The Japanese Orthopedic Association (JOA) score (minimal score −2, maximal score 17) is composed of seven categories (motor function of fingers, shoulder and elbow, and lower extremity, sensory function of upper extremity, trunk, and lower extremity, and bladder function) and is a scoring system that evaluates severity of myelopathy [37]. 

### 5.9. Clinical Factors

To evaluate the clinical factors affecting the effect of BoNT-A injection, clinical variables such as the dystonia pattern (anterocollis or retrocollis), the Kellgren grade for cervical spondylosis, and the injection doses for each muscle were also recorded [38,39]. These clinical factors differed from the primary and secondary outcomes of this study and were analyzed retrospectively.

### 5.10. Safety Measures

All post-injection adverse events were recorded. Discomfort, reduced general condition, headache, dry mouth, injection site pain, neck/shoulder pain, blurred vision, voice alteration, viral infection, dizziness, back pain, tiredness, and inability to lift the head due to weakness were also investigated and recorded. A Video Fluoroscopy Swallowing Study (VFSS) was performed three times (baseline, 12 weeks after the first injection, and 12 weeks after the second injection) for each patient, and additional VFSS was performed when dysphagia occurred after the injection to confirm and identify the abnormal findings.

### 5.11. Statistical Analysis

Statistical analyses were performed using IBM SPSS 20 software (IBM Corporation, Armonk, NY, USA) and SAS. A *p*-value of 0.05 was used as the threshold for statistical significance. A carry-over effect was defined as a significant difference in the TWSTRS score at four weeks between the two treatment sequences [40]. Analysis of variance (ANOVA) was used to examine for a carry-over effect and period effect with changes of TWSTRS total score.

### 5.12. TWSTRS Score

The ANCOVA model was used to compare the changes on the TWSTRS at four weeks and 12 weeks after injections. In this model, the factor was the medication used (BoNT-A versus saline) and the covariate was the baseline value of the TWSTRS. 

### 5.13. NRS Score 

The NRS pain score and NRS disability score were also analyzed using the ANCOVA model. The NRS satisfaction score was analyzed using the Wilcoxon signed-rank test for the comparison of the BoNT-A and saline groups at four weeks and 12 weeks after the injections since baseline satisfaction scores were not applicable for this item. 

### 5.14. Clinical Factors

The ANCOVA model was used to determine whether clinical factors affected the degree of improvement after BoNT-A injection. The degree of improvement was defined as the change in the TWSTRS total score at four weeks. 

## Figures and Tables

**Table 1:** Baseline characteristics and clinical status of the study participants.

Items		Number = 16
Demographics	Male/Female, number	8/8
	Age (years), mean (SD)	46.00 (6.44)
	Retrocollis/Anterocollis, number	7/9
	GMFCS level, number (%)	
	I	4 (25.00)
	II	7 (43.75)
	III	0 (0)
	IV	4 (25.00)
	V	1 (6.25)
	Maximal Kellgren score for cervical spondylosis, number (%)	
	0	0 (0)
	1	2 (12.50)
	2	3 (18.75)
	3	8 (50.0)
	4	3 (18.75)
Clinical data	TWSTRS total score at baseline, mean (SD)	41.69 (13.36)
	TWSTRS Severity score, mean (SD)	20.63 (11.13)
	TWSTRS Disability score, mean (SD)	11.13 (5.66)
	TWSTRS Pain score, mean (SD)	9.94 (4.47)
	JOA score at baseline, mean (SD)	11.34 (2.84)
	NRS pain score at baseline, mean (SD)	4.25 (2.08)
	NRS disability score at baseline, mean (SD)	5.19 (2.46)

TWSTRS, Toronto Western Spasmodic Torticollis Rating Scale; JOA, Japanese Orthopedic Association Score; NRS, Numerical Rating Scale; GMFCS, Gross Motor Function Classification System.

**Table 2:** Treatment site of injection and treatment dose (Number=16).

Injection Site	Number (%)
Sternocleidomastoid	11 (68.8)
Trapezius	6 (37.5)
Levator scapulae	8 (50.0)
Scalenus (medius and anterior)	7 (43.8)
Splenius capitis	7 (43.8)
Longissimus	3 (18.8)
Cervical paraspinalis	2 (12.5)
Splenius cervicis	1 (6.3)

Median dose (range), 145 U (40–200); Mean dose (SD), 139.7 U (50.5); Median number of muscles injected, 3.
